# Design and usability testing of an in-house developed performance feedback tool for medical students

**DOI:** 10.1186/s12909-021-02788-4

**Published:** 2021-06-23

**Authors:** Yadira Roa Romero, Hannah Tame, Ylva Holzhausen, Mandy Petzold, Jan-Vincent Wyszynski, Harm Peters, Mohammed Alhassan-Altoaama, Monika Domanska, Martin Dittmar

**Affiliations:** 1 Quality Assurance in Education, Charité – Universitätsmedizin, Berlin, 10117 Berlin, Germany; 2 Dieter Scheffner Center for Medical Education and Educational Research, Dean’s Office of Study Affairs, Charité – Universitätsmedizin, Berlin, 10117 Berlin, Germany; 3 Business Division IT, Charité – Universitätsmedizin, Berlin, 10117 Berlin, Germany

**Keywords:** Assessment feedback, Usability testing, Formative and summative assessment, Learning analytics, EPAs

## Abstract

**Background:**

Feedback is essential in a self-regulated learning environment such as medical education. When feedback channels are widely spread, the need arises for a system of integrating this information in a single platform. This article reports on the design and initial testing of a feedback tool for medical students at Charité-Universitätsmedizin, Berlin, a large teaching hospital. Following a needs analysis, we designed and programmed a feedback tool in a user-centered approach. The resulting interface was evaluated prior to release with usability testing and again post release using quantitative/qualitative questionnaires.

**Results:**

The tool we created is a browser application for use on desktop or mobile devices. Students log in to see a dashboard of “cards” featuring summaries of assessment results, a portal for the documentation of acquired practical skills, and an overview of their progress along their course. Users see their cohort’s average for each format. Learning analytics rank students’ strengths by subject. The interface is characterized by colourful and simple graphics. In its initial form, the tool has been rated positively overall by students. During testing, the high task completion rate (78%) and low overall number of non-critical errors indicated good usability, while the quantitative data (system usability scoring) also indicates high ease of use. The source code for the tool is open-source and can be adapted by other medical faculties.

**Conclusions:**

The results suggest that the implemented tool LevelUp is well-accepted by students. It therefore holds promise for improved, digitalized integrated feedback about students’ learning progress. Our aim is that LevelUp will help medical students to keep track of their study progress and reflect on their skills. Further development will integrate users’ recommendations for additional features as well as optimizing data flow.

**Supplementary Information:**

The online version contains supplementary material available at 10.1186/s12909-021-02788-4.

## Background

Feedback is an essential element of the educational process [1]. Studies [2, 3] show that good feedback practices may strengthen students’ self-regulative ability. Based on the 7 principles of good feedback practices [2], feedback should: 1) help to define good performance, 2) facilitate self-reflection, 3) deliver high-quality information to students about their learning, 4) encourage teacher and peer dialogue, 5) provide positive motivational beliefs and create self-esteem, 6) close the gap between current and desired performance, 7) provide instructors with information to shape their teaching. In addition to knowledge-based feedback delivered by examinations and grades – the focus during traineeships is on performance-based feedback. In an ideal context, feedback is a continual process between teacher and student. Despite its importance, most (medical) trainees feel that they do not receive adequate feedback, and when they do, the process is not effective [4]. This is in line with similar observations [5] which point to the fact that often there is no appropriate time and place for feedback sessions in medical training. However, effective feedback also affects metacognition, self-directed learning and self-awareness, and these competencies are in turn important for medical students’ learning progress [6].

The necessity for the development of a feedback tool for the modular curriculum of medicine (MCM) became clear over the course of several accreditation procedures and evaluations of the degree programme by students, who expressed the need for reliable and structured feedback on their learning progress. In recent years, the Charité has piloted projects to introduce online feedback for medical students. An e-portfolio system offering a variety of features to stimulate learning was trialed using Wordpress (Avila et al., 2016 [7]). A later EU cooperation with 16 other universities aimed to combine feedback from Entrustable Professional Activities (EPAs, [8, 9] together with assessment data using Learning Analytics (LA) into an e-portfolio for workplace-based assessment (Holzhausen et al., 2019 [10]). Due to both the specific nature of the Charité medical curriculum in addition to the cost factor of purchasing external software, the faculty opted to create an in-house tool. The aim was to integrate assessment results, both summative (legally binding, determining whether a necessary standard has been reached) and formative (primarily to guide learning), together with EPA evaluations, and using learning analytics, provide feedback to students on their individual progress.

Software usability is key to achieving acceptance and making an impact on the target learner population, [11–15]. Maintaining a strong user-centered design approach during development helps to maximize usability [16, 17]. Based on this evidence, we ensured that the development and implementation process saw a strong, early focus on user needs. In the following we report on the design and usability testing of the new feedback tool.

## Implementation

### Development process and prototype testing

**Fig. 1:**
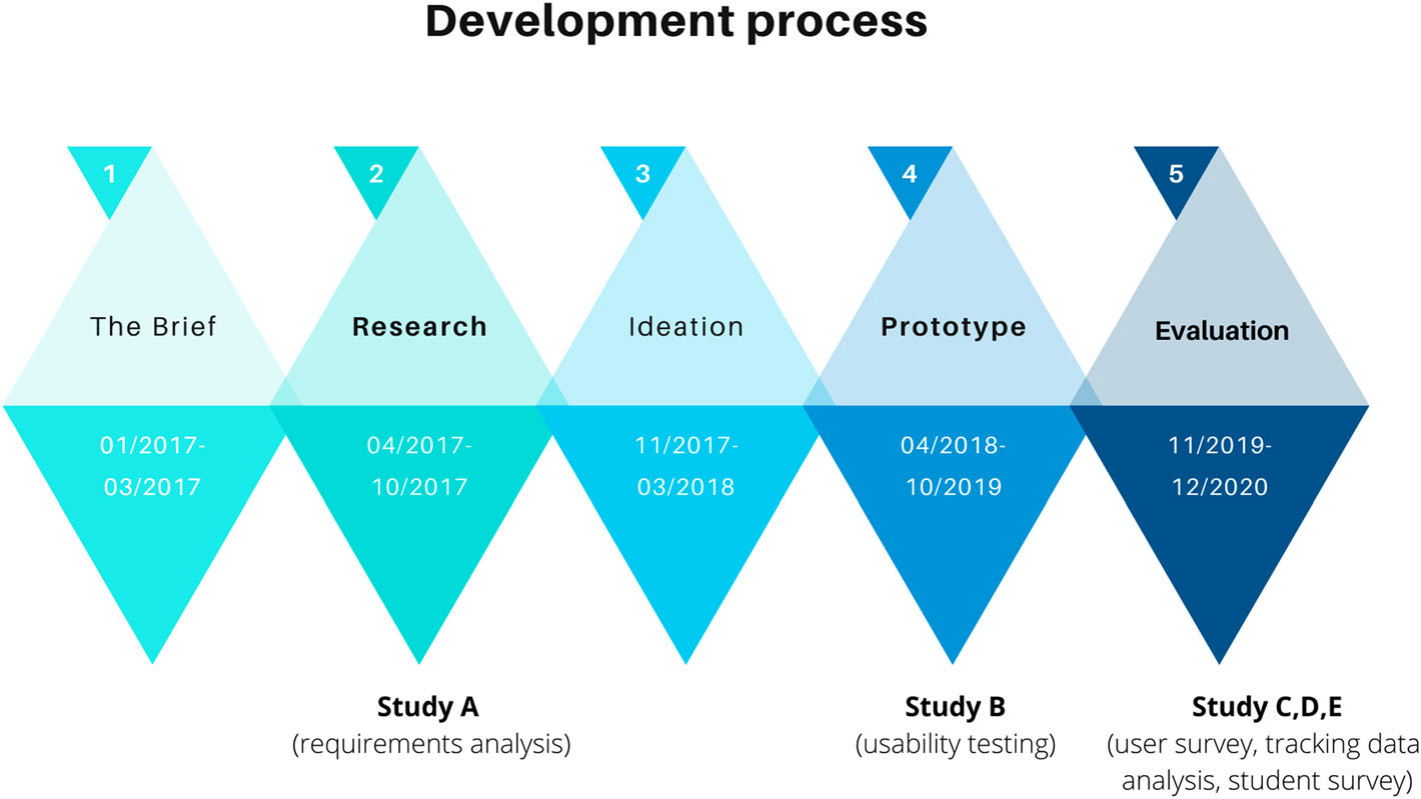
Development timeline for LevelUp

The development of the feedback tool LevelUp was characterized by iterative feedback loops between technical developers, project managers and medical student users at each development stage (Fig. 1). In the following, we elaborate on studies conducted as part of the research and prototyping stages, specifically a requirements analysis (study A) and usability testing (study B).

#### Study A) Requirements analysis (research stage)

An online requirements analysis questionnaire was conducted to identify student needs for better feedback on their learning progress (attachment A). This was sent to students (2nd semester and above) on the undergraduate medical curriculum at the Charité in 2017 (total *n* = 2974). Students were asked if they wanted more feedback on their learning progress and if they would use a feedback tool for this purpose (5-point scale ranging from 1 = strongly agree to 5 = strongly disagree). In addition, a categorical item elicited students’ opinions on preferred features and functions.

#### Requirements analysis results

**Fig. 2:**
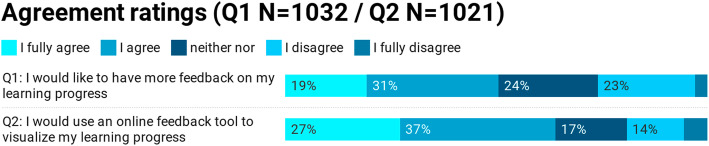
Study A) Result of students’ ratings in the requirements analysis regarding the need for feedback and expected use of a feedback tool

**Fig. 3:**
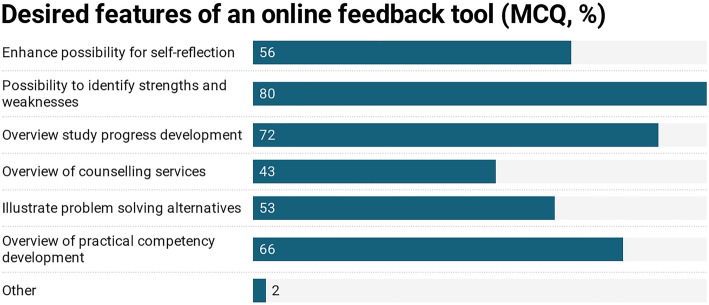
Study A) Desired features for a feedback tool, student responses

In total, 1032 students participated (response rate = 34%, mean age of 25 (SD = 4), 65% female). 50% of students expressed a desire for more feedback on their learning progress (M = 2.6; SD = 1.1) and 63% were interested in using a feedback tool which incorporated formative and summative feedback (M = 2.3; SD = 1.2, see Fig. 2). In response to the question of which features an online feedback tool should contain, 80% of students wanted a mode to identify their strength and weaknesses, 72% a comprehensive overview of all exam results, and 66% more feedback on their progress in clinical skills (see Fig. 3).

Based on the results of study A, we firstly designed paper prototypes for the new tool. Medical students were continuously involved in the development of several prototype versions to ensure a good match to their needs. Once a satisfactory prototype had been created, programming began on a digital prototype.

### Technical development following paper prototyping

The front end of the tool was programmed in React and Redux, using ReactJS as a framework. The back end is covered by a PHP-based application using the Symfony framework and a relational database, where assessment results are imported, computed and stored in the same way as user actions.

Data security and confidentiality was an important aspect in designing the platform. It is crucial that only students can access their own data and that even in case of a data breach, no personal data can be accessed. We achieved this by not saving any personal data on the live server. Instead, we create personal hashes on a separate internal server, which has access to all personal data. On the live server, all assessment results are connected only to the personal hash. The hash can only be re-created directly after login with the personal data given by the identity provider. A data protection concept for the development of the tool, which also covered usability testing during the pilot phase of the project, was approved by the office for data protection at the Charité.

In the following we describe the testing of the prototype tool with medical students.

#### Study B) Usability testing (prototype & iteration stage)

This study followed the commonly recommended approach of using multiple usability measures and collected both subjective, self-reported data as well as objective data obtained by recording and analyzing the interactions of medical students with the software (attachment B).

The development team conducted the test using a prototype laptop version of LevelUp. Camtasia software (TechSmith) captured the participant’s voice (audio input), comments, navigation choices and the click paths, in a think aloud approach.

Twenty-two medical students (mean age 23.8 years, 14 females (63%) participated, who were equally distributed among semesters 2–11. Prior to completing the tasks, participants were asked about their expectations for a feedback tool. During the session, the test administrator explained the testing procedure and asked the participant to complete a brief background questionnaire. During the test, the task completion rates (in %), time on task (in s.) and the number of non-critical errors were recorded (non-critical errors are errors that do not prevent successful completion of the task and the scenario). Overall satisfaction with the tool and the experience were measured by qualitative feedback at the end of the usability test.

#### Statistical analysis

All statistical analyses were carried out using SPSS 25 [18]. Descriptive statistics show mean (M) and standard deviation (SD).

#### Results of usability testing

##### Background information

The interviews revealed that 96% of students wanted more feedback on their learning progress (*N* = 21) and that 86% already used commercial and non-commercial online self-assessment tools (*N* = 19) to compensate this lack of feedback.

##### Task completion success rate

Averaged over all tasks and participants, the task completion success rate was 78%. Many tasks were carried out successfully (see Table 1). Notable was the very low completion rate (5%) for Task 10, which required students to find a number of professional activities listed in the area of EPAs.

**Table 1:** Overview of usability test data over 12 tasks. SD = standard deviation

Averaged task completion rate and time on task (*N* = 22)			
Task	Task Completion	Time on Task (sec) +/− SD	Avg. Number of errors +/− SD
1	91%	60 +/− 30	0.45 +/− 0.96
2	100%	40 +/− 24	0.10 +/− 0.31
3	82%	61 +/− 33	0.64 +/− 0.90
4	100%	15 +/− 13	0 +/− 0
5	73%	58 +/− 29	1.32 +/− 1.20
6	73%	37 +/− 18	0.95 +/− 0.97
7	91%	24 +/− 15	0.36 +/− 0.66
8	91%	22 +/− 21	0.32 +/− 0.57
9	77%	67 +/− 35	1.41 +/− 1.82
10	5%	37 +/− 25	0.9 +/− 0.44
11	55%	39 +/− 27	0.91 +/− 0.53
12	100%	23 +/− 17	0.22 +/− 0.52

Some tasks were inherently more difficult to complete than others, which is reflected by the average time spent on each task. On average, participants spent 40 s (SD = 24) on the completion of each task. The number of errors depends on the complexity of the task. Participants made the most non-critical errors in task 9 and 5. Task 4 showed the least number of errors.

##### Redesign following study B

The usability test identified 2 main issues based on participants’ completion rates and time on task. The first was a lack of understanding of both the theoretical concept and implementation of the EPA feature as presented on the tool. Participants showed reduced completion rates for both EPA tasks, and comments revealed a need for better orientation. The EPA-feature redesign focused on resolving these issues. We firstly reduced the complexity of the metrics, using 3 main scores distinguished by spacing and colour. The second issue addressed a more general problem of information architecture and graphic visualizations on the prototype tool, and we concluded that the navigation click paths needed simplifying. We redesigned the navigation structure by introducing “cards” on the dashboard page, and enhanced the discoverability of call-to-action buttons. We also reduced the complexity of graphs and bar charts. Following redesign the tool was released to all students.

##### Usability evaluation following release – methods

The final design of LevelUp was evaluated using surveys following release. Two questionnaires addressed usability of the tool amongst both LevelUp users and students who had not yet used the platform.

##### Study C

Since January 2020, registered users have had the opportunity to take part in an online survey to evaluate their experiences with the platform (attachment C), accessed exclusively via a link on the dashboard page. A questionnaire including standard usability and website experience measurement criteria, the System Usability Scale (SUS) [19], a 7-point Likert scale ranging from 1 = strongly agree to 7 = strongly disagree) was chosen. The 7-point scale selected for psychometric reasons [20] was converted to 5 points for the SUS calculation. In order to assess the intention to revisit the website [21], further items were applied. These questions were set in rating scale format (7-point Likert scale ranging from 1 = strongly agree to 7 = strongly disagree). A single item (5-point scale ranging from 1 = strongly agree to 5 = strongly disagree) assessed the overall impression of the platform [22].

##### Study D

From January – March 2020, we evaluated students’ experiences with LevelUp via an additional online questionnaire (attachment D) sent out to all MCM students. Two dichotomous single choice questions determined whether students were already aware of the tool and had used it. Respondents were classified as ‘user’, or one of two types of ‘non-user’: ‘type 1 non-user’ (aware of tool) and ‘type 2 non-user’ (not yet aware of tool). Based on this, students were asked open-ended questions to address the features they liked most and to elicit recommendations or improvements for LevelUp (user group), as well as general expectations and desired features for a feedback tool (non-user group).

A quantitative and qualitative analysis of open-ended questions on the current features, suggested improvements as well as obstacles to using LevelUp was carried out. The resulting comments were evaluated with regard to their main themes, and further categorized qualitatively into sub-themes. The overall number of comments, and comments relating to specific categories, were also assessed quantitatively to rank themes in order of priority.

## Results: tool release and evaluation

**Fig. 4:**
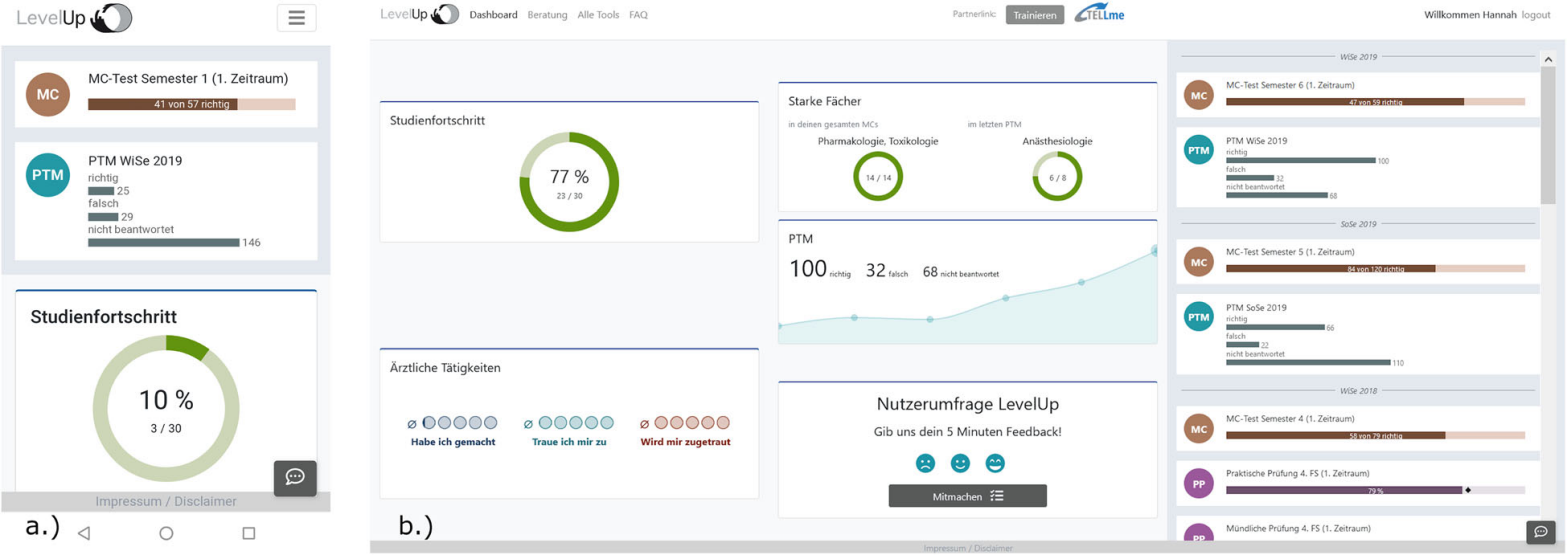
LevelUp dashboard screenshot **A** mobile device **B** desktop

The final design for LevelUp was released for all medical students in November 2019. The feedback tool is a browser app for desktop or mobile. Students log in using their student email address and matriculation number. LevelUp is currently available in German and a demonstration version can be viewed at https://levelup.charite.de. On signing in, the user is presented with a personal dashboard page displaying clickable feature cards, from which the tool can be navigated (see Fig. 4). In addition to graphic visualizations of all assessment results, including markers for the average of their cohort, users are able to garner the following feedback on their progress:

- Detailed multiple choice (MCQ) exam results including feedback on all questions and answers with solutions
- Detailed visualization of their knowledge growth with the aid of the Progress Test Medicine (PTM) feature
- A personal checklist of requirements fulfilled to progress onto the next stage of their course
- A review of acquired practical competencies (EPAs) to assist students in preparing for their medical clerkships. Students can enter self-evaluations in the form of a score on a supervision scale for individual professional activities, as well as request evaluations from their teachers
- Personal strengths ranked according to subject area

The specific features of LevelUp are detailed in Table 2.

**Table 2:** Overview of features

LevelUp features	
Feature	Description
Dashboard	Visually tracks, analyzes and displays performance indicators and key data points to monitor progress
Study Progress Tracker	Overview of study progress visualizing milestones (e.g. clerkships) and completed modules and exams
Timeline	Chronological overview of all completed assessments
Detailed MCQ Exam results	Visualization of MCQ performance in comparison to student cohort, subject and module, with statistical details
Strengths and weaknesses analysis	Ranking of strengths and weaknesses according to subject area, based on MCQ exams and PTM
Practical competencies (EPAs)	Records the development of practical competencies incl. self-assessment and evaluation by teachers
Longitudinal overview of PTM results	Overview of longitudinal and cross-sectional PTM performance per subject
Advice services	An overview of all advice services for students at the Charité
Online Tools	Links to all useful tools and platforms for students at the Charité

In the following we report on evaluation results following initial release (studies C, D and E).

### Study C): Dashboard survey

Responses were analyzed in March 2020 and again in April 2021. A sample of 22 users (semesters 2–9) gave feedback via the dashboard survey in the initial analysis. The overall impression of the platform LevelUp was rated positively (M = 1.8, SD = 0.9) by all participants. Students’ intention to use and revisit rating (M = 2.2, SD = 1.3) showed that students have a strong intention to revisit the platform. The mean SUS rating of 81.2 (SD = 14.1) indicated a good usability of the platform LevelUp (a “good” SUS score is above 76 [23, 24]). In April 2021 we were able to re-analyze with 68 responses resulting in a mean SUS score of 79.5 (SD = 15.1), intention to revisit as 2.5 (SD = 1.5) and an overall rating of 2.0 (SD = 0.9).

### Study D): Broad LevelUp evaluation survey

**Fig. 5:**
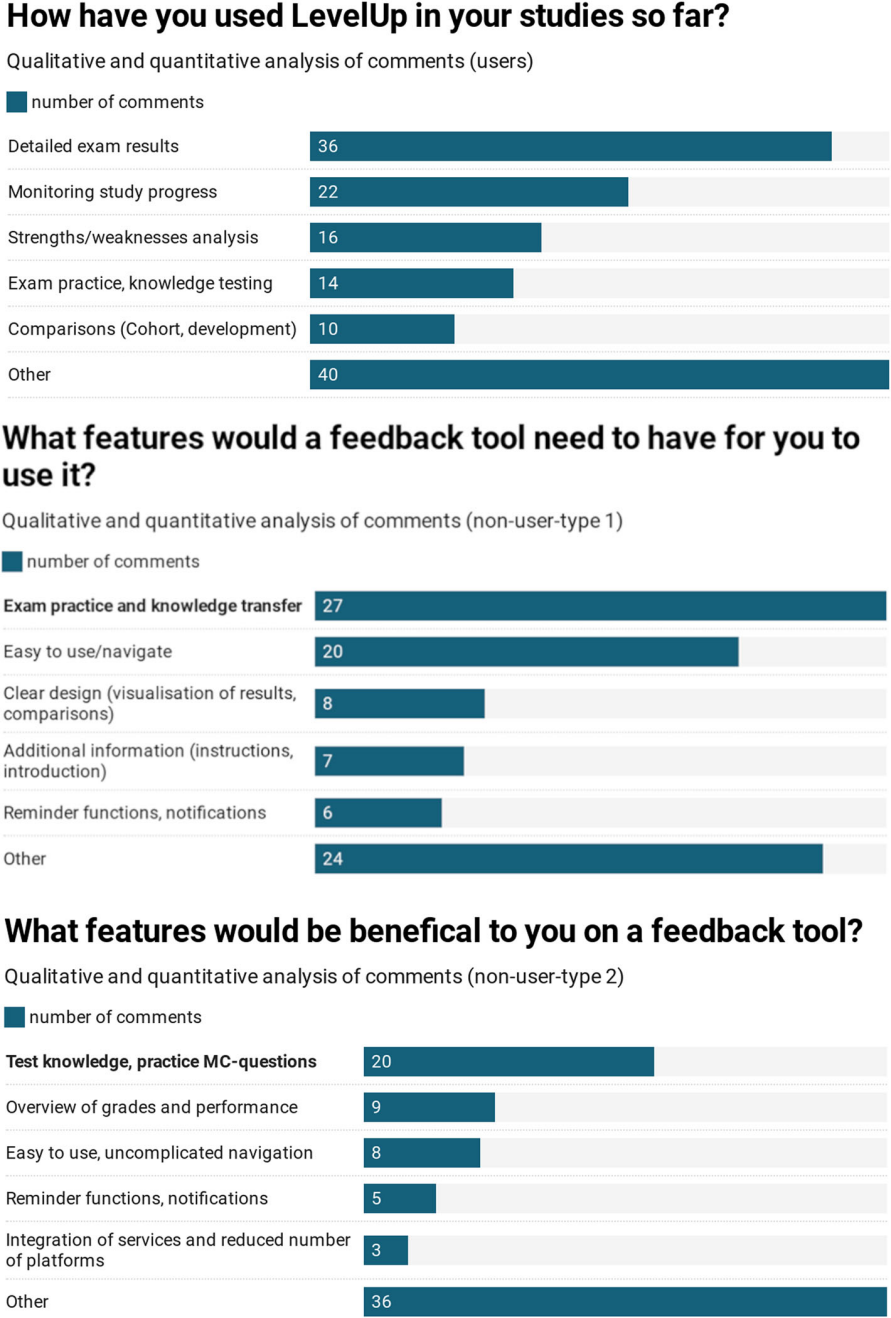
Study D) Broad student survey - results of qualitative commentary analysis with the main topics for the user group, non-user group type 1 and non-user group type 2

In total a sample of 736 students participated (response rate = 22%,=24.7 years (SD = 4.6 years), female = 64.6%, diverse =1.6%). In total, 223 students (30%, user group) produced 138 responses to the open-ended question “How do you currently use LevelUp?”. Two hundred forty-three students (33%, non-users type 1 group) generated 92 responses overall according to the open-ended question “What features does a feedback tool need”. Finally, 270 students (37%, non-users type 2 group) made 81 comments in response to the question “What features would be beneficial to you in a feedback tool?” (see Fig. 5).

### Study E) Web analytics

Matomo open-source web analytics software is used to analyze traffic on the platform, including clicks on each feature. Student registrations on the platform are documented, as well as the number of requests to login-restricted features, which serves as a measurement of the frequency of use. In line with data protection regulations, this data cannot be assigned to specific users.

The number of registrations to the tool from semesters 1–10 is currently 2219 (N total = 3639). This number increases significantly with each new semester. According to Matomo, approx. 139,300 actions (clicks) by registered users were tracked in LevelUp between release in November 2019 and April 2021. Up to April 2021, there were a total of 36,450 visits, with users spending an average of 2 min on the site, and the average number of actions per visit 8.

## Discussion

### Interface usability

The applied forms of usability testing (study B-D) indicated an overall ease of use and positive evaluation of the new platform. Features which were perceived to be difficult to understand were adapted and redesigned. Web analytics (study E) showing a high number of page visits since release indicate that students are using the new platform. The SUS-score of 79.5 suggests that LevelUp can be regarded as acceptable within the acceptability range (70–100%). This score has also been associated with a verbal user rating of ‘good’ (score 72–85) according to other studies [24, 25]. However, the primary use of the SUS is to classify the ease of use of a website, it is not a diagnostic tool for identifying areas of improvement [21, 23, 25, 26]. The qualitative comments from the survey (D) reiterate the high SUS-score.

### Value as a feedback tool and further development

The majority of comments (study D) highlight that having a centralized site to find integrated formative and summative feedback about their study progress is key for most participants. The overall concept of LevelUp specifically focuses on the facilitation of self-reflection by collating and analyzing assessment results together with self-evaluations on a single platform. The study progress tracker, the statistical details on MCQ assessments and the PTM-feature give detailed information to students about their learning. LevelUp thus adheres to several principles of good feedback [2].

Improvements still need to be made to the tool. In particular, involving teachers and instructors in the feedback process will be a priority in the further development of the tool. Teachers could for instance use LevelUp to obtain feedback about their teaching, evaluations, assessments or MCQs they have created. An increase of bidirectional feedback may strengthen the general feedback culture within the faculty. LevelUp does not currently offer a function for qualitative data entry such as text and commentary, which would be a necessary further development if the above feature is to be realized.

There is evidence in the qualitative survey data that non-users who were not yet aware of LevelUp would appreciate features (e.g. study progress tracker and self-evaluation of practical competencies) that are already provided by LevelUp. Based on these findings, a current strategy to promote LevelUp through a wide range of channels (e.g. social media, posters) aims to boost registrations.

Further studies are needed to investigate the effects of the feedback tool in groups of regular users and non-users amongst undergraduate medical students over the course of their degree and the effect on e.g. reducing exam drop-out rates. Registrations to the tool increase with each semester and we expect new students to grow used to using LevelUp throughout their studies. More senior students may be less inclined to use the tool due to its late introduction. The EPA feature becomes more relevant for senior students preparing for their clerkships, hence investigating usability for this target group and communicating the value of the new tool to senior students will be the focus of future iterations. Web analytics Matomo gathers information on the number of clicks on each feature, and analysis of this data over time will guide development of the tool. Students’ recommendations for improvements will be garnered as part of a broad student survey in late 2021, continued analysis of the dashboard survey responses, as well as qualitative interviews to be carried out on campus. Optimizing the speed of data flow into the tool from assessments will further increase the tool’s usefulness.

#### Usability study limitations

The usability study has limitations. Regarding Study C, in total 30 (including former paper prototype testing) students were involved in this process. This limits the generalizability of the results for larger student cohorts. Our results may also be limited by a selection bias due to the voluntary nature of the testing. The qualitative and quantitative survey data (B, D) yielded a much larger sample, arguably providing greater reliability.

Matomo web analytics software provides valuable statistics on visits to the tool. However, in line with data protection regulations, these statistics cannot be assigned to individual users. This means that we do not currently have sufficient information on particular groups of students, such as semester cohorts, and how they use the tool. A planned broad student survey in late 2021 will yield information on use of the platform per semester group.

#### LevelUp as an adaptable, open-source software

The source code for the platform was released under an open source licence in May 2020 and can be downloaded free at Github (see software availability). Institutions wishing to implement feedback software can adapt the system to meet their requirements with relatively few resources. The current implementation is not necessarily specific for the Charité, but specific for the given assessment formats. If, for example, MCQ tests with mapped tags such as subjects are a given assessment format, the data structure can already be used by other universities.

The aim of the open-source project is to create a more standardized and customizable software. Other faculties would nevertheless need to create import adapters for integrating the data of their specific assessment formats into the data structure of LevelUp. Currently, the user interface is only available in German, but can easily be translated.

## Conclusions

The results of the usability test and online survey suggest that in its early release form, LevelUp is well-accepted and fulfils a need for better feedback for students on the modular curriculum at the Charité. Students appreciate having an overview of the complete range of assessment results presented to them on a single, easy to use platform. The good usability of the tool means that it is likely students will return to the platform regularly. Data from repeated usability testing as well as broader student evaluation surveys will provide input for its continued development. Our long term aim for the tool is that it will provide high-quality online feedback for students as part of a strategy to close a perceived gap in feedback during their medical studies.

## Availability and requirements

**Project name:** LevelUp

**Project home page:** https://levelup.charite.de

**Operating system(s):** web-based application (Firefox, Chrome, Safari, MS-Edge)

**Programming language:** Front end: React and Redux, using ReactJS as a framework. Back end: PHP-based application using the Symfony framework and a relational database.

**License:** GNU Affero General Public License v3.0 https://github.com/charite-studium-und-lehre

**Any restrictions to use by non-academics:** none

## Supplementary Information

**Additional file 1.** Attachment A. Study A, general student survey 2017 (excerpt, questions on learning progress and feedback).

**Additional file 2.** Attachment B. Study B, pre-interview and usability tasks for LevelUp.

**Additional file 3.** Attachment C. Study C (dashboard user-questionnaire based on the System Usability Score (SUS).

**Additional file 4.** Attachment D. Study D, general student survey 2020 (excerpt, questions on the newly released LevelUp).

## Data Availability

The data used and analyzed during the current study are available from the corresponding author upon reasonable request.

## Abbreviations

MCM: Modular Curriculum of Medicine, the degree in medicine at Charité - Universitätsmedizin, Berlin
MCQ: Multiple choice questions
PTM: Progress Test Medicine
EPAs: Entrustable Professional Activities
SD: Standard deviation
SUS: System usability scale
