# Supplementary material for: Design and usability testing of an in-house developed performance feedback tool for medical students
Source: BMC Med Educ. 2021 Jun 23;21:354. doi: 10.1186/s12909-021-02788-4 (PMC8220763; doi:10.1186/s12909-021-02788-4)
Supplement: Supplementary file 1 — Additional file 1. Attachment A. Study A, general student survey 2017 (excerpt, questions on learning progress and feedback). [file 12909_2021_2788_MOESM1_ESM.docx]

# Attachment A

Study A, general student survey 2017 (excerpt, questions regarding learning progress and feedback)

1. In which semester on the Modular Curriculum in Medicine are you studying?

2. What is your gender?

*Male/Female/Diverse*

3. I am satisfied with my learning progress over the last semester.

*Fully agree - 1 2 3 4 5 - Fully disagree*

4. I would like to receive more feedback on my learning progress.

*Fully agree - 1 2 3 4 5 - Fully disagree*

5. I would be willing to use an online feedback tool which visualizes my individual learning progress

*Fully agree - 1 2 3 4 5 - Fully disagree*

(If you are willing to give us feedback in the form of a short interview on the development of the feedback tool, please click on the email address below. Thank-you for your support.)

6. An online feedback tool on individual learning progress should include the following features:

- The possibility of self-reflection
- The possibility to identify strengths and weaknesses
- An overview of my learning progress development (PTM, course exams)
- Show advice services in case of problems
- Show alternative problem solving strategies (learning strategies, learning tips)
- Overview development of my practical skills
- Other

7. Which other features do you think an online tool should have?

*(Answer entered as a comment)*
