# Supplementary material for: Design and usability testing of an in-house developed performance feedback tool for medical students
Source: BMC Med Educ. 2021 Jun 23;21:354. doi: 10.1186/s12909-021-02788-4 (PMC8220763; doi:10.1186/s12909-021-02788-4)
Supplement: Supplementary file 2 — Additional file 2. Attachment B. Study B, pre-interview and usability tasks for LevelUp. [file 12909_2021_2788_MOESM2_ESM.docx]

# Attachment B

# Study B, Pre-interview and usability tasks for LevelUp

## Pre-interview

1. In which semester are you studying?
2. Gender:
3. Age:
4. Do you use Amboss?
5. Do you know your exam results from HIS (Hochschulinformationssystem/University Information System)? *Yes, No, Partly*
6. 🡪 If not, why not?
7. Do you use your examination results on HIS? *Yes, No, Partly*
   - If not, why not
8. Have you used your results from the progress test medicine? *Yes, No, Partly*
9. How have you used your results from the progress test medicine?
10. Which values (Correct, Incorrect, Not answered, Certain, Probable, Guessed) do you use from the progress test for your self-evaluation?
11. I would like more feedback on my learning progress. *Yes, No, Why not?*
12. To what extent are you able to rate your current individual strengths and weaknesses on your medical studies? *Yes, No, Why not?*
13. What expectations do you have for the functions of the LevelUp feedback tool?

## Usability tasks

1. How do you rate your progress on your course as a whole when you look at the dashboard view “course progress”? Do you have any missed attendances or semester exams (if so, in which semester)?
2. Do you see a need to take action when looking at your data? What steps do you need to take?
3. What percentage (and score) did you achieve in the semester exam (3rd semester)?
4. In which module in the 3rd semester did you achieve the highest score?
5. How many difficult questions did you answer correctly in your 3rd semester exam?
6. What was your best Progress Test Medizin result (please state the semester)?
7. In which part (clinical ward) of the ward exam in the 4th semester did you achieve the best percentage?
8. The ward can be divided into different task areas. In which task area did you achieve the best result?
9. Which were your three strongest subjects in the semester examinations across all semesters and three weakest subjects in the last PTM?
10. How many professional activities are listed in the area “care of patients”?
11. How many ratings can be entered in the area of „communication with patients“?
12. How many supervision levels do you have for the procedure „taking venous blood“ for the section „I have done this“?
