# Supplementary material for: Design and usability testing of an in-house developed performance feedback tool for medical students
Source: BMC Med Educ. 2021 Jun 23;21:354. doi: 10.1186/s12909-021-02788-4 (PMC8220763; doi:10.1186/s12909-021-02788-4)
Supplement: Supplementary file 3 — Additional file 3. Attachment C. Study C (dashboard user-questionnaire based on the System Usability Score (SUS). [file 12909_2021_2788_MOESM3_ESM.docx]

# Attachment C

# Study C (Dashboard user-questionnaire based on the System Usability Score (SUS)

1. I declare that I am participating in this survey voluntarily.
2. In which semester are you studying in on the Modular Curriculum of Medicine (not counting vacation semesters)?
3. How often do you use LevelUp when preparing for examinations (number of times accessed in a month)?
4. How often do you use LevelUp during course time (number of times accessed in a month)?
5. I think that I would like to use this system frequently.

*Fully agree - 1 2 3 4 5 6 7 - Fully disagree*

1. I found the system unnecessarily complex.

*Fully agree - 1 2 3 4 5 6 7 - Fully disagree*

1. I thought the system was easy to use.

*Fully agree - 1 2 3 4 5 6 7 - Fully disagree*

1. I think that I would need the support of a technical person to be able to use this system.

*Fully agree - 1 2 3 4 5 6 7 - Fully disagree*

1. I found the various functions in this system were well integrated.

*Fully agree - 1 2 3 4 5 6 7 - Fully disagree*

1. I thought there was too much inconsistency in this system.

*Fully agree - 1 2 3 4 5 6 7 - Fully disagree*

1. I would imagine that most people would learn to use this system very quickly.

*Fully agree - 1 2 3 4 5 6 7 - Fully disagree*

1. I found the system very cumbersome to use.

*Fully agree - 1 2 3 4 5 6 7 - Fully disagree*

1. I felt very confident using the system.

*Fully agree - 1 2 3 4 5 6 7 - Fully disagree*

1. I needed to learn a lot of things before I could get going with this system.

*Fully agree - 1 2 3 4 5 6 7 - Fully disagree*

1. I will use the platform again.

*Fully agree - 1 2 3 4 5 6 7 - Fully disagree*

1. I will use the platform regularly.

*Fully agree - 1 2 3 4 5 6 7 - Fully disagree*

1. I would recommend the platform to friends and family.

*Fully agree - 1 2 3 4 5 6 7 - Fully disagree*

1. If I am interested in these topics in the future, I think I would use the platform again.

*Fully agree - 1 2 3 4 5 6 7 - Fully disagree*

General impression

1. Overall I would give the platform the grade…

*Very good – Good – Satisfactory – Sufficient/Pass – Insufficient/Fail*

1. What do you especially like about the tool? *(Answer as a comment)*
2. What suggestions for improvement of the tool do you have? *(Answer as a comment)*
