# Supplementary material for: Design and usability testing of an in-house developed performance feedback tool for medical students
Source: BMC Med Educ. 2021 Jun 23;21:354. doi: 10.1186/s12909-021-02788-4 (PMC8220763; doi:10.1186/s12909-021-02788-4)
Supplement: Supplementary file 4 — Additional file 4. Attachment D. Study D, general student survey 2020 (excerpt, questions on the newly released LevelUp). [file 12909_2021_2788_MOESM4_ESM.docx]

# Attachment D

# Study D, general student survey 2020 (excerpt, questions regarding the newly released LevelUp)

1. In which semester of the MCM are you currently studying?
2. What is your gender?

*Male/Female/Diverse*

1. Have you heard of LevelUp?

*Yes or No*

1. Have you used LevelUp?

*Yes or No*

Open questions for users (Yes), *answers entered as a comment*:

1. How have you used LevelUp recently for your studies?
2. What obstacles did you encounter in using the tool?
3. What other features would be beneficial to you in LevelUp?
4. How should feedback be given on your course in order for it to be helpful to you?
5. What kind of feedback on your progress would you like to receive on your course?
6. What features of a feedback app would be beneficial to you in your studies?

Open questions for non-users (No), *answers entered as a comment*:

1. What have you heard about LevelUp?
2. What would you need on a study progress feedback app to make you want to use it?
3. What would prevent you from using a study progress feedback app?
